# Supplementary material for: Diet Impacts on Gene Expression in Healthy Colon Tissue: Insights from the BarcUVa-Seq Study
Source: Nutrients. 2024 Sep 16;16(18):3131. doi: 10.3390/nu16183131 (PMC11434945; doi:10.3390/nu16183131)
Supplement: Supplementary file 1 [file nutrients-16-03131-s001.zip › nutrients-3183011-supplementary/SupplFiguresTableS1.docx]

**SUPPLEMENTARY TABLES AND FIGURES**

**SUPPLEMENTARY FIGURES**

**Supplemental Figure S1. Correlation plots of dietary patterns and food group variables adjusted by the residual method by sex.**

1. **Male**
2. **Female**


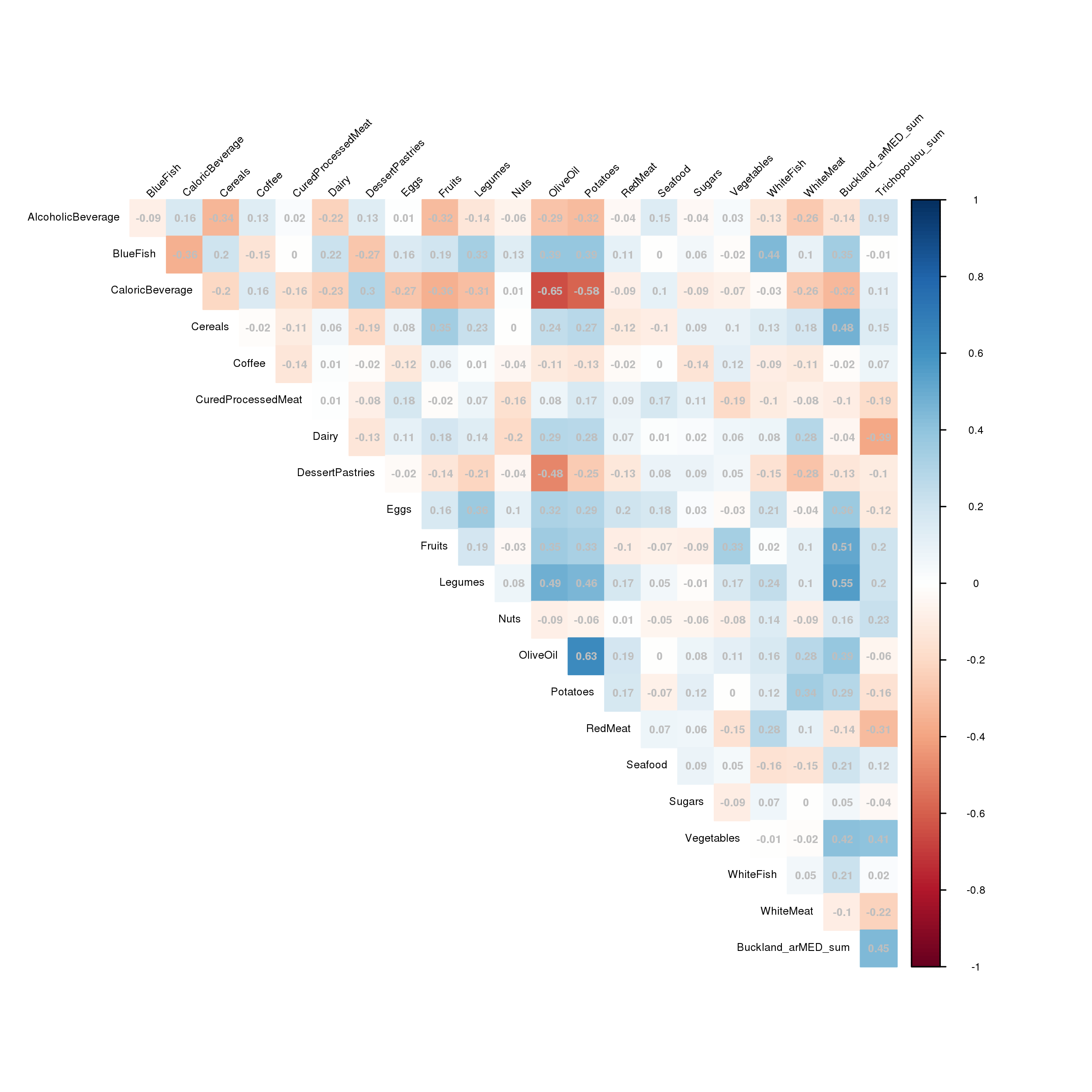

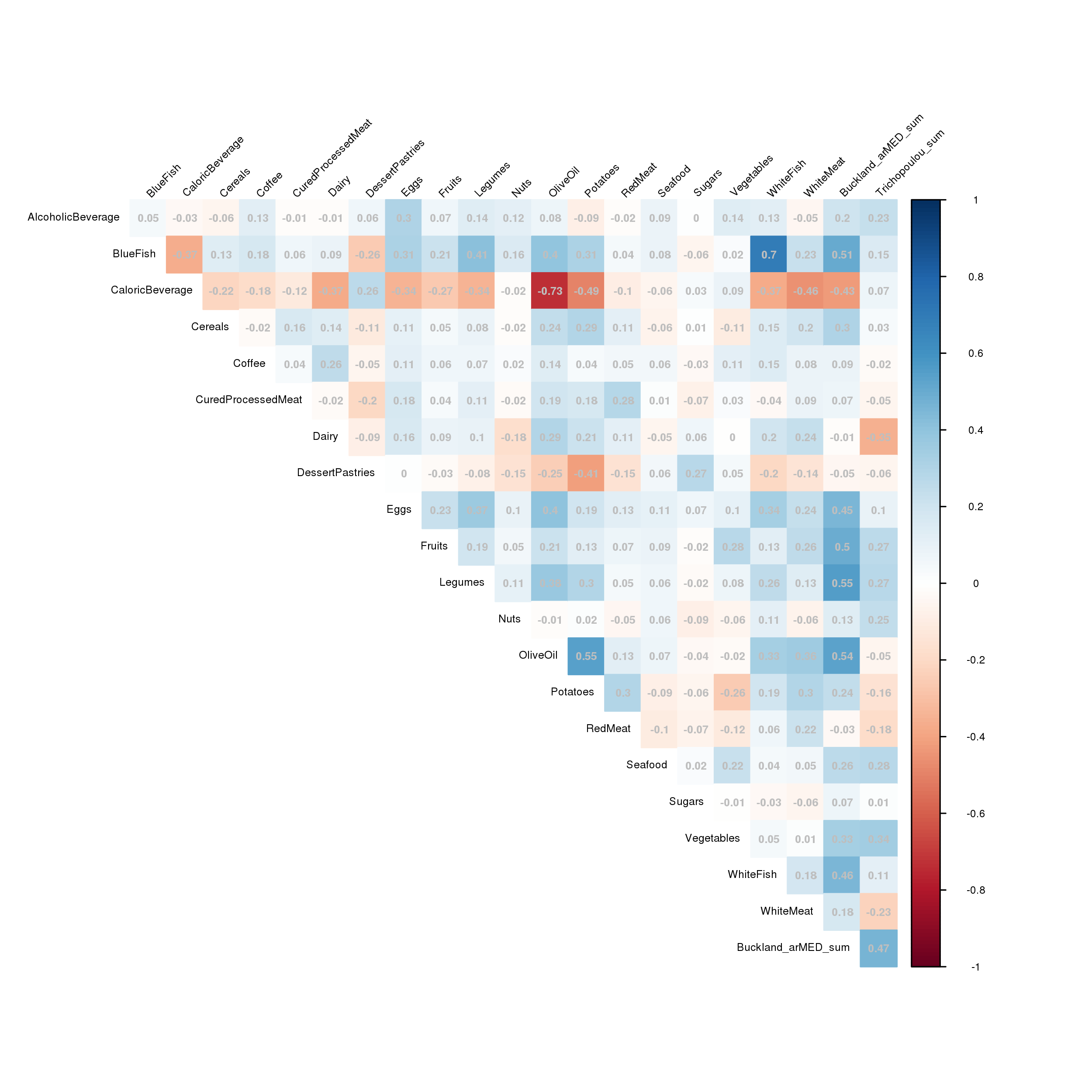


**Supplemental Figure S2. Protein-Protein Interaction Network for the food group blue fish.**

**
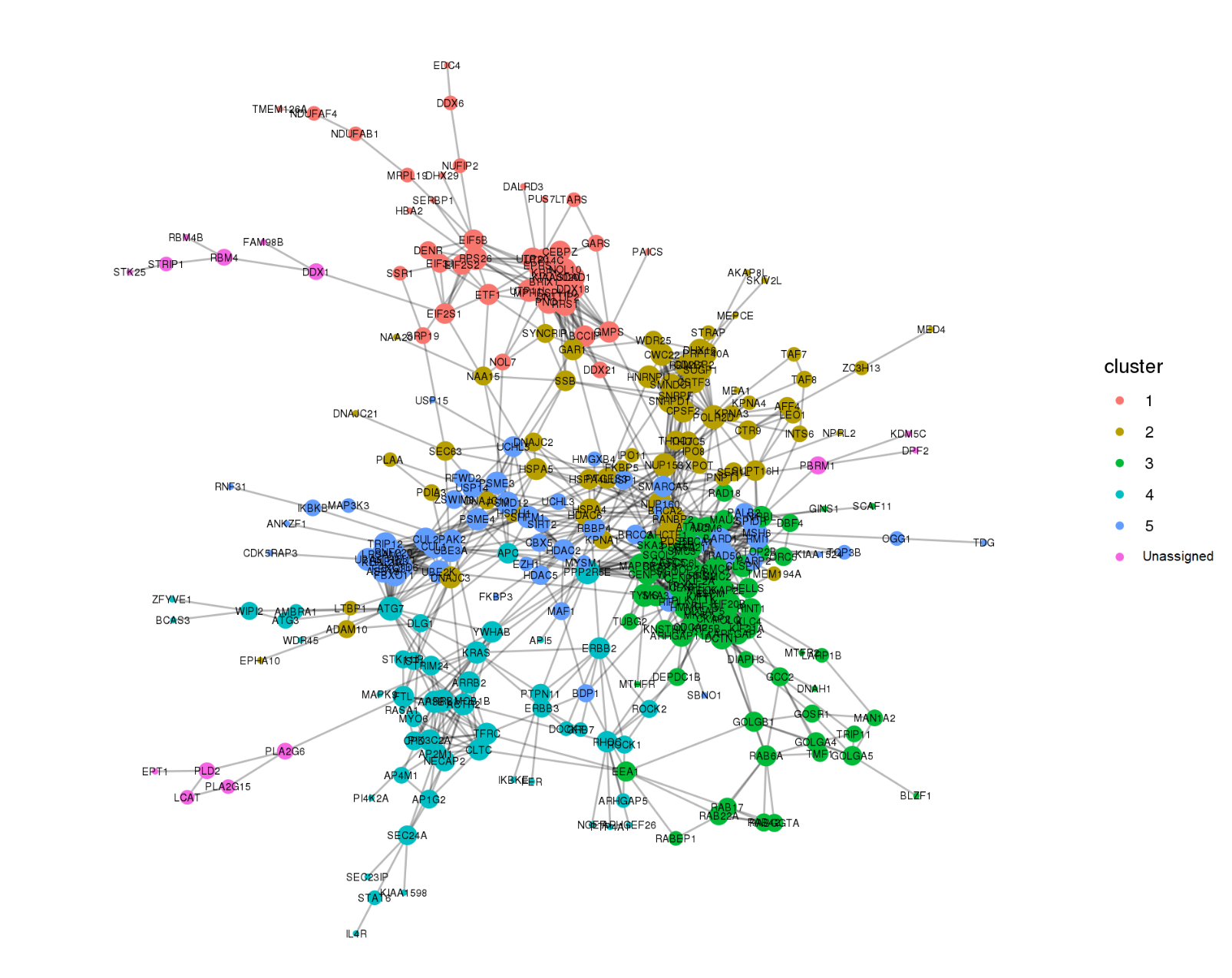
**

Nodes represent protein coding genes colored by network clusters. Clusters without associated enriched terms labelled as unassigned.

**Supplemental Figure S3. Protein-Protein Interaction Network for the food group alcoholic beverages.**


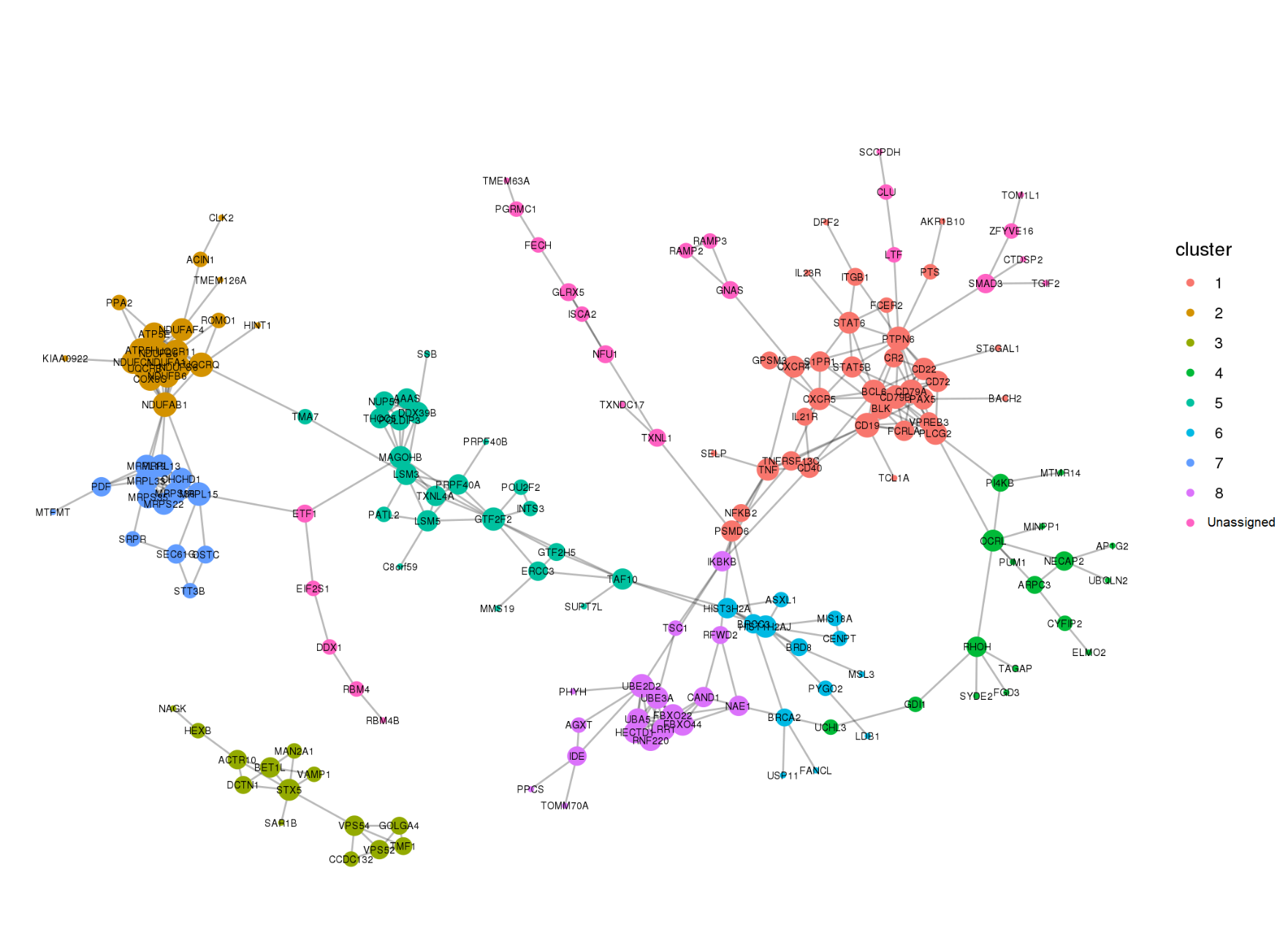


Nodes represent protein coding genes colored by network clusters. Clusters without associated enriched terms labelled as unassigned.

**Supplemental Figure S4. Protein-Protein Interaction Network for the food group potatoes.**

**
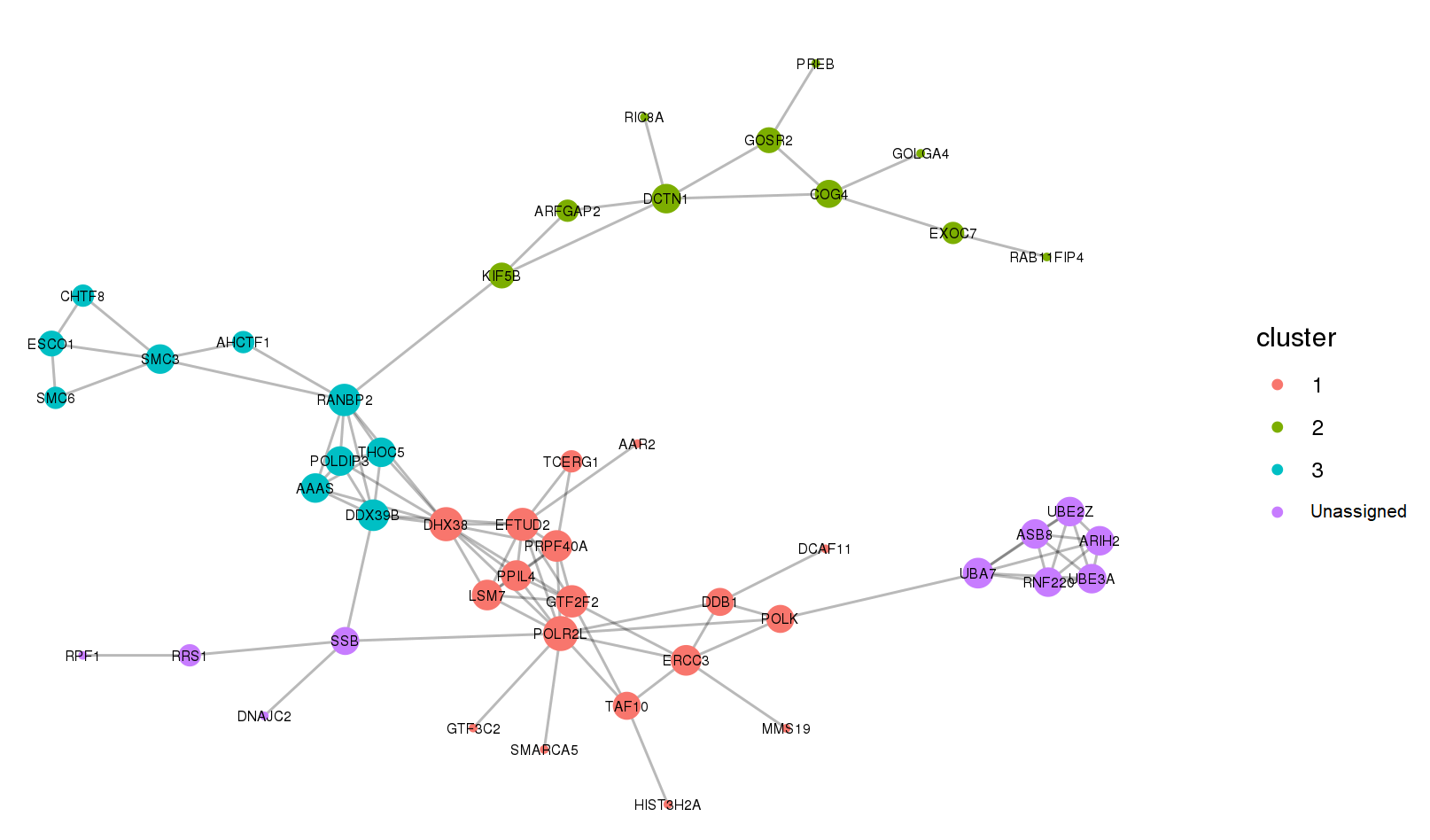
**

Nodes represent protein coding genes colored by network clusters. Clusters without associated enriched terms labelled as unassigned.

**SUPPLEMENTARY TABLES**

| **Supplemental Table S1. Number of differentially expressed genes across all dietary variables by false discovery rate value.** | | | | |
| --- | --- | --- | --- | --- |
|  | **Number of differentially expressed genes** | | | |
|  | **Main analysis** | | **Sensitivity Analysis** | |
| **Food groups** | **FDR<0.05** | **FDR<0.1** | **FDR<0.05** | **FDR<0.1** |
| Potatoes | 2586 | 3605 | 226 | 553 |
| Caloric beverages | 1786 | 2754 | 10 | 372 |
| Olive oil | 987 | 1694 | 5 | 5 |
| Blue fish | 538 | 1097 |  |  |
| Alcoholic beverages | 402 | 1155 |  |  |
| Dessert pastries | 68 | 263 |  |  |
| Dairy | 48 | 109 |  |  |
| Coffee | 37 | 150 |  |  |
| Vegetables | 16 | 39 |  |  |
| White fish | 13 | 268 |  |  |
| Sugars | 6 | 11 |  |  |
| Red meat | 5 | 5 |  |  |
| White meat | 3 | 31 |  |  |
| Nuts | 3 | 3 |  |  |
| Legumes | 2 | 2 |  |  |
| Eggs | 1 | 1 |  |  |
| Fruits | 1 | 1 |  |  |
| Sea food | 0 | 1 |  |  |
| Cured and processed meat | 0 | 0 |  |  |
| Cereals | 0 | 0 |  |  |
| Mediterranean-Diet Score | 0 | 18 |  |  |
| *adapted-relative* Mediterranean Diet Score | 0 | 0 |  |  |
| FDR: false discovery rate | | | | |

**Supplemental Table S2. Sensitivity analysis in the top 5 food groups additionally adjusted by BMI.**

Please see the enclosed Excel file.

**Supplemental Table S3. List of differentially expressed genes in the top 5 food groups adjusted by the residual method (FDR < 0.05).**

Please see the enclosed Excel file.

**Supplemental Table S4. Results of Reactome based functional analysis for the food group blue fish.**

Please see the enclosed Excel file.

**Supplemental Table S5. Results of Reactome based functional analysis for the food group alcoholic beverages.**

Please see the enclosed Excel file.

**Supplemental Table S6. Results of Reactome based functional analysis for the food group potatoes.**

Please see the enclosed Excel file.

**Supplemental Table S7. Results of functional Protein-Protein Interaction (PPI) network analysis for the food group blue fish.**

Please see the enclosed Excel file.

**Supplemental Table S8. Results of functional Protein-Protein Interaction (PPI) network analysis for the food group alcoholic beverages.**

Please see the enclosed Excel file.

**Supplemental Table S9. Results of functional Protein-Protein Interaction (PPI) network analysis for the food group potatoes.**

Please see the enclosed Excel file.
